# 935. Flash Forward: Bright Ideas to Improve Burn Photography and Image Documentation

**DOI:** 10.1093/jbcr/irag033.502

**Published:** 2026-04-06

**Authors:** Adrienne E DeVault, Mary E Bruce, Marisa Jones, Taylor Champagne, Anjay Khandelwal

**Affiliations:** Akron Children's Hospital, Akron, OH; Akron Children's Hospital, Akron, OH; Akron Children's Hospital, Akron, OH; Akron Children's Hospital, Akron, OH; Akron Children's Hospital, Akron, OH

## Abstract

**Introduction:**

High-quality clinical photography is a critical component of burn documentation. Clear, well-composed images allow for accurate assessment of injury severity, donor site availability, healing progression, and graft take. These images also serve as legal documentation and may be requested by patients or families as part of the medical record. However, suboptimal images—due to poor lighting, distracting backgrounds, or incorrect angles—can misrepresent both the injury and the quality of care provided. Recognizing the variability in image quality within our institution, we conducted a retrospective review and launched a quality improvement (QI) initiative aimed at standardizing and enhancing the photographic documentation of burn patients.

**Methods:**

A six-month retrospective chart review was performed on admissions to a regional burn center. Exclusion criteria included patients admitted directly from the operating room or discharged within 48 hours. A nine-point standardized scoring rubric was developed to evaluate image quality, assessing both background interference and foreground clarity. Standards were established for photograph timing (admission, post-burn day 2, and discharge) and completion of two electronic burn distribution diagrams per patient. Following baseline data collection, an educational intervention was delivered to clinical staff focusing on photographic technique, consistency, and clinical utility. A structured QI process was then implemented.

**Results:**

Seventy-nine patient charts were reviewed. Images averaged 4.0/9 for background and 4.16/9 for foreground quality. While 99% of charts included admission photographs, only 33% contained images at all three critical timepoints. Additionally, 53% lacked a second electronic burn diagram, despite an average TBSA change of 3.59% between diagrams. These findings suggest significant documentation gaps that could impact clinical assessment and continuity of care. Post-intervention data indicates a positive impact of education and standardized protocol implementation on image and documentation quality.

**Conclusions:**

Photographic and diagrammatic documentation in burn care is often inconsistent and suboptimal. A targeted education session and performance improvement strategy can enhance the quality, consistency, and clinical utility of these essential visual records. Standardizing practices improves not only care coordination and assessment accuracy but also fulfills medico-legal and patient record responsibilities.

**Applicability of Research to Practice:**

High-quality patient imagery supports clinical decision-making, continuity of care, and accurate outcomes tracking. Integrating structured education into staff workflows fosters sustained quality improvements and aligns documentation practices with modern standards in burn care.

**Funding for the study:**

N/A.

